# Combining Continuous Smartphone Native Sensors Data Capture and Unsupervised Data Mining Techniques for Behavioral Changes Detection: A Case Series of the Evidence-Based Behavior (eB^2^) Study

**DOI:** 10.2196/mhealth.9472

**Published:** 2018-12-10

**Authors:** Sofian Berrouiguet, David Ramírez, María Luisa Barrigón, Pablo Moreno-Muñoz, Rodrigo Carmona Camacho, Enrique Baca-García, Antonio Artés-Rodríguez

**Affiliations:** 1 Department of Psychiatry and Emergency Brest Medical University Hospital Brest France; 2 IMT Atlantique Lab-STICC F-29238 Brest France; 3 SPURBO EA 7479 Université de Bretagne Occidentale (UBO) Brest France; 4 CHRU Cavale Blanche University Hospital of Brest Brest France; 5 Universidad Carlos III de Madrid Leganés Spain; 6 Gregorio Marañón Health Research Institute Madrid Spain; 7 Department of Psychiatry Fundación Jiménez Díaz Hospital Madrid Spain; 8 Department of Psychiatry Autónoma University Madrid Spain; 9 Centro de Investigación en Salud Mental Carlos III Institute of Health Madrid Spain

**Keywords:** behavioral changes, data mining, mental disorders, sensors, wearables

## Abstract

**Background:**

The emergence of smartphones, wearable sensor technologies, and smart homes allows the nonintrusive collection of activity data. Thus, health-related events, such as activities of daily living (ADLs; eg, mobility patterns, feeding, sleeping, ...) can be captured without patients’ active participation. We designed a system to detect changes in the mobility patterns based on the smartphone’s native sensors and advanced machine learning and signal processing techniques.

**Objective:**

The principal objective of this work is to assess the feasibility of detecting mobility pattern changes in a sample of outpatients with depression using the smartphone’s sensors. The proposed method processed the data acquired by the smartphone using an unsupervised detection technique.

**Methods:**

In this study, 38 outpatients from the Hospital Fundación Jiménez Díaz Psychiatry Department (Madrid, Spain) participated. The Evidence-Based Behavior (eB^2^) app was downloaded by patients on the day of recruitment and configured with the assistance of a physician. The app captured the following data: inertial sensors, physical activity, phone calls and message logs, app usage, nearby Bluetooth and Wi-Fi connections, and location. We applied a change-point detection technique to location data on a sample of 9 outpatients recruited between April 6, 2017 and December 14, 2017. The change-point detection was based only on location information, but the eB^2^ platform allowed for an easy integration of additional data. The app remained running in the background on patients’ smartphone during the study participation.

**Results:**

The principal outcome measure was the identification of mobility pattern changes based on an unsupervised detection technique applied to the smartphone’s native sensors data. Here, results from 5 patients’ records are presented as a case series. The eB^2^ system detected specific mobility pattern changes according to the patients’ activity, which may be used as indicators of behavioral and clinical state changes.

**Conclusions:**

The proposed technique could automatically detect changes in the mobility patterns of outpatients who took part in this study. Assuming these mobility pattern changes correlated with behavioral changes, we have developed a technique that may identify possible relapses or clinical changes. Nevertheless, it is important to point out that the detected changes are not always related to relapses and that some clinical changes cannot be detected by the proposed method.

## Introduction

### Data Capture in Patient Environment

Web-based and smartphone apps offer new opportunities for patient monitoring. The integration of these tools into medical practice has heralded the electronic health (eHealth) era. eHealth involves the integration of new technologies into routine clinical practice by increasing networking possibilities between patients and clinicians. Recent trials using mobile electronic devices have proven successful in real-world and real-time monitoring and have improved the assessment possibilities in a large panel of clinical settings [1]. The assessment of patients’ dynamic relationships between events and disease course is enhanced by the development of momentary data collection strategies such as experience sampling methods and ecological momentary assessment (EMA). These approaches, which rely on delivering informative contents and self-administered questionnaires, reduce the recall bias, as they are done in quasi-real time, but these face many limitations, including poor data reliability, burden and intrusiveness for patients, and data security issues [2].

In addition, electronic devices can perform passive (or autonomous) data gathering, that is, to extract information about users without any effort on their part. Actigraphy, geolocation, and communication activity are usual features of current smartphones and may be indicators of patients’ behavior if they are properly processed. Advances in sensors technology and novel textile-electronic integration techniques also draw new perspectives for behavior ecological assessment. Moreover, it is currently possible to find commercially available wearable sensing technologies for several wellness and clinical purposes: simple heart rate monitors [3], rehabilitation after surgical intervention [4], and monitors of physical activity or sleep quality assessment [5]. Overall, an extensive panel of physical and mental conditions (eg, insomnia, diabetes, problems associated with older age, cardiac problems, or respiratory problems) can be remotely monitored by appropriate health care professionals—physicians, doctors, or nurses. These devices are often connected to a smartphone, which increases the networking capabilities and the user experience. Furthermore, the collected data can be processed and transferred over the internet to a remote clinical backend server for further analysis, assessment, and decision making and intervention if needed.

### Monitoring Activities of Daily Living and Mobility Patterns

The emergence of smart homes and wearable sensor technologies allows nonintrusive collection of activity data [6]. Thus, health-related events, such as activities of daily living (ADLs; eg, feeding and sleeping) and patients’ mobility patterns, can be captured without their active participation [7]. Monitoring behavioral changes of psychiatric patients and their ability to carry out their ADLs will likely improve the knowledge about the disease course. For example, the detection of changes in behavioral patterns may help in detecting emerging disorders [8]. In addition, smart home and ambient assisted living systems use sensors and other devices that are either wearable or integrated in the patients’ home and have been used to assess the effect of undesirable symptoms and cognitive impairment on ADL functions [9] or to detect emerging disorders based on changes in patients’ behavior [10]. The ease of access to smartphone technology for the general population and recent technological advances in smartphone-integrated sensors are paving the way for behavioral changes detection, based only on activity assessment. Physical activity assessment is usually based on findings from brief, regularly scheduled, in-person appointments or self-reported questionnaires [11]. Although widely used, this approach reduces the assessment in cross-sectional observations that miss essential information and are subject to recall bias. In this study, the data obtained from smartphones and integrated devices will be processed to identify mobility pattern changes, as they may be correlated with behavioral changes and clinical changes. For example, an increase in depressive symptoms is associated with a reduction of the patients’ physical activity [12]. Thus, patient’s mobility patterns may be used as proxies for behavioral changes. In a clinical setting, the detection of mobility pattern changes could be used by clinicians or caregivers as signals of (possible) behavioral changes in their patients.

Along the lines proposed in this work, recent studies have shown that smartphone data can be used to identify behavioral changes in patients. Abdullah et al. [13] reported that combining self-reported data with data from several smartphone sensors and communication patterns resulted in the reliable prediction of the Social Rhythm Metric, a clinically validated marker of stability and rhythmicity for individuals with bipolar disorder. Another system, Monsenso, collects and extracts voice features from phone calls that were made during everyday life in naturalistic settings [14]. Concretely, the MONARCA II Research Project, which uses Monsenso, obtained 6552 numerical features related to the pitch and voice variance that were extracted from patients’ phone calls during their everyday life. Another platform is Beiwe, which is a research-oriented platform for digital phenotyping. Using Beiwe, Barnett et al. [15] developed a method to predict schizophrenia based on anomaly detection.

Considering the strengths and pitfalls of smartphone monitoring strategies, we have designed a system capable of performing continuous monitoring of patients using the smartphone and wearable sensors and data entry (data from phone calls, messages, and so on). This Evidence-Based Behavior (eB^2^) platform comprises a smartphone app, which collects these data, and a backend server, which stores and processes them. The eB^2^ app collects data from inertial sensors, physical activity, phone calls and message logs, app usage, nearby Bluetooth and Wi-Fi connections, and location. In addition, using Google Play Services, the app can access detailed activity information and nearby location data. Moreover, wearable devices provide information like the body temperature, heart rate, or galvanic skin response. The app was developed to run in the background, and users only interact with the app for the initial configuration. Furthermore, it was designed with battery-safe considerations like noncontinuous recording schedule, automatic sleep and wake function, and it additionally notifies the operating system to relaunch itself when it is closed or stopped because of users’ actions or failures and reboots.

### Hypothesis and Principal Objective

We hypothesize that it is feasible to develop an analysis method capable of detecting mobility pattern changes based on the data acquired by the eB^2^ system. Moreover, we believed that these changes might serve as proxies for behavioral changes. This study aims to assess the feasibility of detecting mobility pattern changes in a sample of outpatients using a smartphone app and an unsupervised detection method, which was run on a backend server.

## Methods

### Summary

We performed an unsupervised detection method and a qualitative analysis of a sample of 5 patients out of 38 outpatients enrolled in the eB^2^ study between April 6 and December 14, 2017. The eB^2^ study was (and still is) a 2-year, multicenter-controlled trial conducted by the Fundación Jiménez Díaz. Concretely, it was a prospective study that aimed to determine whether the behavioral changes detected by the eB^2^ system correlated with any clinical change. Note, however, that in this preliminary work, we only focused on mobility pattern changes.

### Participants

Patients who received psychiatric care in an outpatient mental health center of the Psychiatry Department at Fundación Jiménez Díaz, a University Hospital in Madrid, Spain, were approached to participate in this study. This department is part of the National Health Service and provides medical coverage financed by taxes to a catchment area of 420,000 people. The research followed the Code of Ethics of the World Medical Association (Declaration of Helsinki).

### Patient Inclusion and Exclusion Criteria

The inclusion criteria for this study were either male or female outpatients aged ≥18 years, diagnosed with mood disorders (ICD-10 codes F30-F39) or adjustment disorders (ICD-10 code F43.2), and coping with depression. Moreover, patients had to own a smartphone with an Android or iOS operating system, be connected to a Wi-Fi network, at least, once a week, and had to have given written informed consent for the eB^2^ study. Participants were excluded if they were under the age of 18 years, illiterate, enrolled in other trials, or were in situations that did not allow obtaining written informed consent. Participants were not paid. Members of the study office (EBG, MLB, and RC) established an initial list of patients that met the inclusion criteria. The contents of the monitoring interviews were reviewed to identify patients who had attended, at least, 2 appointments. These criteria yielded the aforementioned 38 outpatients.

### Research Protocol

**Figure 1:**
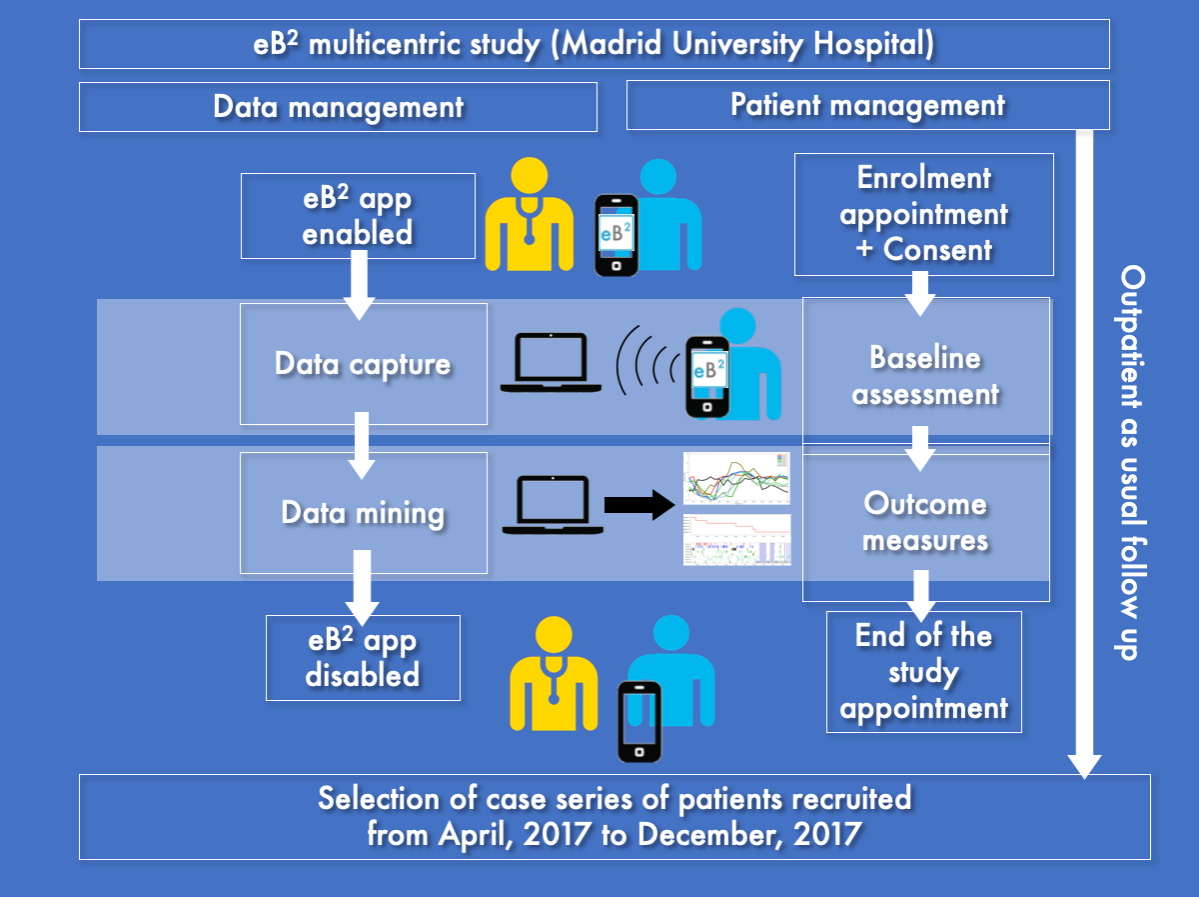
Visual representation of the study protocol. eB^2^: Evidence-Based Behavior.

The eB^2^ app was downloaded on patients’ smartphones on the day of recruitment and configured with the assistance of a physician (Figure 1). The app remained running in the background in patients’ smartphones during the study. As previously pointed out, the app was designed with no patient interface, that is, no action of patients was required to capture data.

The eB^2^ app collected the following data: actigraphy, global positioning system (GPS) location, Google location, app usage log, phone calls and message logs, nearby Wi-Fi and Bluetooth devices, and inertial measurement unit signals. The data gathered by the eB^2^ app was anonymized if it was sensitive data (position and phone numbers), then it was translated to a unique data schema, and finally transmitted via Wi-Fi to the eB^2^ backend server where it was stored. The transmission was done through a RESTful application programming interface (API), which had been developed using the JAVA Spring framework. This API is secure sockets layer protected and, to restrict access to the patients’ information, a token-based access policy was implemented following the OAuth2 standard.

In addition to the data captured by the smartphone app, it was possible to collect data provided by third-party APIs, which were also translated into the common data schema by a service that ran at the server. The authorization to use these APIs was requested from the smartphone app and was also token-based. Moreover, data from wearable devices, like Fitbit or Microsoft Band 2, could also be uploaded. It is important to point out that, in this study, only the GPS location was used, which resulted in a simple technique and allowed for an easy clinical interpretation. Finally, signal processing and machine learning algorithms treated the acquired data to extract information, which was used afterwards by clinicians.

### Baseline Characteristics

The baseline characteristics were recorded during an in-person interview for 38 patients enrolled in the eB^2^ study. Variables collected for each patient profile were sex, age, Patient Health Questionnaire-9 (PHQ-9) score [16], diagnosis, and treatment. Clinical diagnoses were made by psychiatrists and coded according to the ICD-10 for mental disorders. Moreover, in each appointment, a psychiatrist administered the PHQ-9 questionnaire, which was designed to assess depression. These variables were entered manually into a secured electronic health record. Each patient was identified by a numeric code to ensure patient anonymity; this code was stored in the database and remained the same throughout all contact with patients. This study did not include a control group.

### Outcome Measures

The principal outcome measure of this study was the identification of changes in patients’ mobility patterns based on the smartphone’s sensors data, which were processed by an unsupervised detection technique. We postulated that these changes could correlate with behavioral changes and relapses. That is, mobility patterns changes were proxies for (more general) behavioral changes. These data were interpreted for each selected patient in the light of the clinical data gathered in routine appointments during study participation.

### Description of the Unsupervised Detection Technique

The proposed unsupervised detection technique comprised 2 algorithms. The first one was an unsupervised clustering technique that defined types of days. This classification was done according to the mobility profile, which was also learned in an unsupervised fashion. The mobility profiles could show, for instance, whether a patient was more active in the morning, afternoon, or evening, or even not active at all. The first step of the clustering technique was to summarize the measured distance acquired on an interval of a few minutes into larger 1-hour intervals and then the aggregated distances were stacked into 24-dimensional vectors; that is, each of these vectors corresponded to a given day, and each component was the cumulative distance traveled by the patient in the corresponding hour. Once we had these vectors, a clustering technique based on a mixture of Gaussians [17] was applied. The parameters of the model, that is, the means and covariance matrices (which we assumed diagonal with only 2 different values out of the 24 possible) were estimated using the Expectation-Maximization algorithm [17]. In particular, the estimated mean of each cluster defined what we called mobility profile, as it showed that in the corresponding cluster, the patient was more active (traveled more distance during the day, night, or at commuting hours). In addition, the Expectation-Maximization algorithm also allowed the handling of missing data, which corresponded to hours for which location data were not available. The final comment regarding the clustering step is the selection of the number of clusters. That is, the allowed number of different profiles (or types of days). This selection obviously depended on the amount of available data, that is, more data allowed the technique to learn more profiles properly. However, an incorrect choice (too large or too small) would result in poor performance. Hence, we used an automatic method, which was based on the minimum description length (MDL) criterion [17].

Regardless of whether a patient was stable or not, these profiles were likely to change from day to day owing to weekends or public holidays. Hence, to detect mobility pattern changes, it did not suffice to detect profile changes (from one type of day or cluster to another). Concretely, we needed to detect changes in the distribution of these profiles. As an example, for a stable patient, the most likely profile was that of a workday, and a different profile could have appeared for the weekends. Nevertheless, the transition from one to another was not identified as a change. What we had to detect was, for instance, if these workday profiles started to appear less often because the patient stopped going to his or her work. Hence, we applied a change-point detection technique to identify when the probability (a portion of time) of each type of day suddenly changed. Moreover, this change-point detector could handle missing data. Then, the clustering technique handled missing hours, and the change-point detector handled missing days.

The technique described so far only exploited information given by the traveled distance, but both the technique and the eB^2^ system may be generalized to incorporate other types of data. For instance, we may exploit how many phone calls were made every hour and, similar to the distance traveled profiles, we should detect changes in the distribution of these calls. Nevertheless, in this preliminary study, we wanted to study the feasibility of this detection based only on the traveled distance, as it resulted in a simple technique that was easier to interpret.

## Results

### Summary of the Results

**Figure 2:**
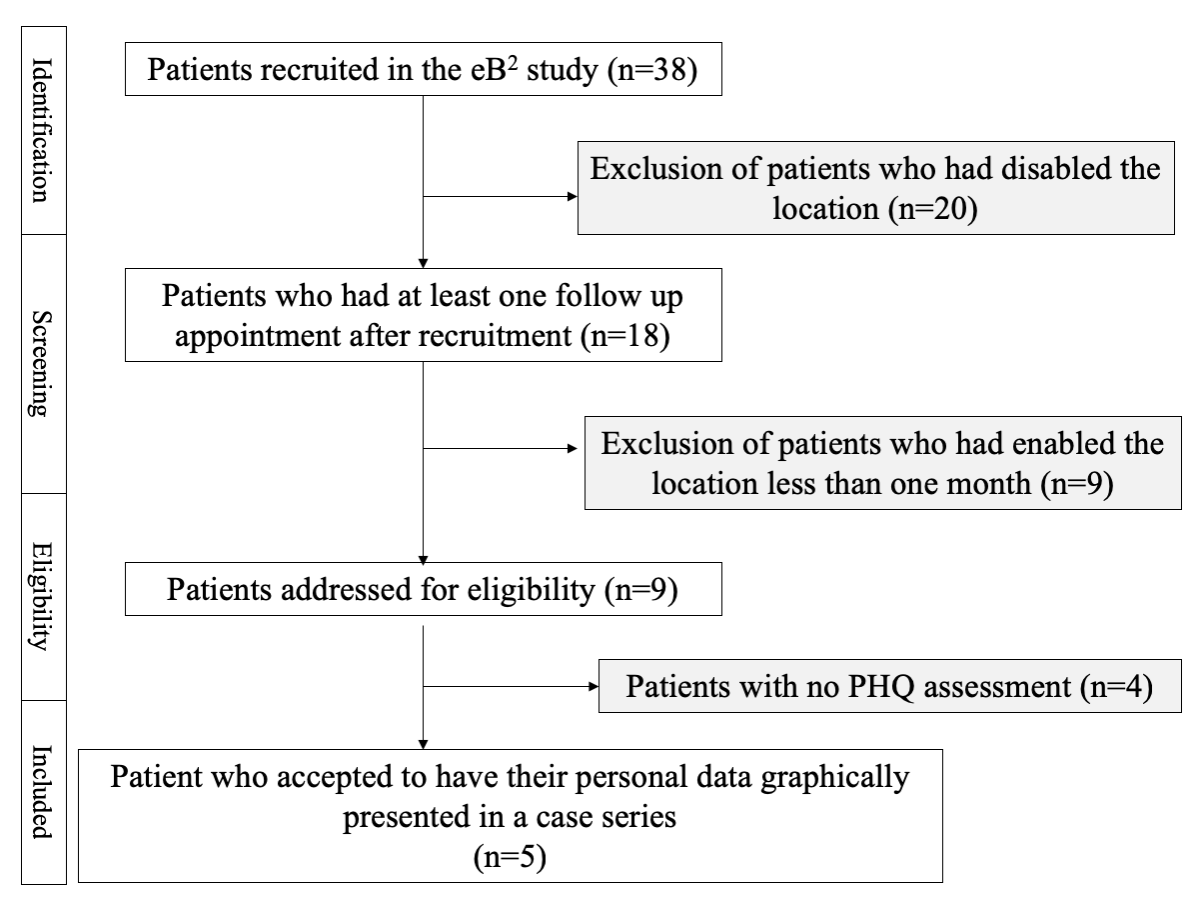
The patient selection process. eB^2^: Evidence-Based Behavior; PHQ: Patient Health Questionnaire-9.

Figure 2 presents the patient selection process of the case series. In the eB^2^ study, 38 patients were recruited when we started the patient selection process for this case series. Nevertheless, of these 38 patients, only 18 had enabled the location (GPS), and of these 18, only 9 had the location enabled for >1 month, which was approximately the required time for the technique to work properly. That is, during the study, many patients disabled the location.

We addressed 9 patients for eligibility. Table 1 summarizes the results for those 9 patients, showing the number of monitored days, number of profiles or clusters, number of detected change-points, number of days between change-points, and the phone model and operating system version.

**Table 1:** Statistics of patients addressed for eligibility.

Patient	Monitored days (n)	Profiles (n)	Detected change-points (n)	Days between change-points (n)	Phone model-operating system
A	323	8	5	17, 35, 95, 54, 9, 113	Samsung Galaxy S7-6.0.1
B	240	5	0	N/A^a^	Samsung Galaxy A5-6.0.1
C	233	4	1	154, 79	Samsung Galaxy A5-7.0
D	162	4	0	N/A	Samsung Galaxy J7-6.0.1
E	75	3	1	49, 26	BQ Aquaris M5-6.0.1
F^b^	155	4	1	37, 118	Sony Xperia M5-6.0
G^b^	222	9	2	6, 30, 186	Huawei Y6-5.1
H^b^	154	5	3	33, 8, 47, 66	Samsung Galaxy J7-6.0.1
I^b^	41	3	0	41	Samsung Grand Prime-5.1.1

^a^N/A: not applicable.

^b^Patient not presented in the case series.

In the following, to shed some light on the technique and results, we present more detailed results for patients A-E as a case series, which were the patients to whom the PHQ-9 questionnaire was administered during routine appointments.

### Detailed Analysis of Five Selected Patients

Patient A was a 56-year-old woman. She was diagnosed with recurrent depressive disorder and fibromyalgia. She was prescribed a daily oral medication of duloxetine 90 mg, quetiapine 150 mg, pregabalin 300 mg, and zolpidem 10 mg. She had regular bedtime and wake-up times during the study period. The clinical assessment of depression showed high scores of PHQ-9: 21 on April 6, 2017, and 25 on May 31, 2017. Unfortunately, this woman dropped out of medical follow-up, and there are no more clinical assessments.

**Figure 3:**
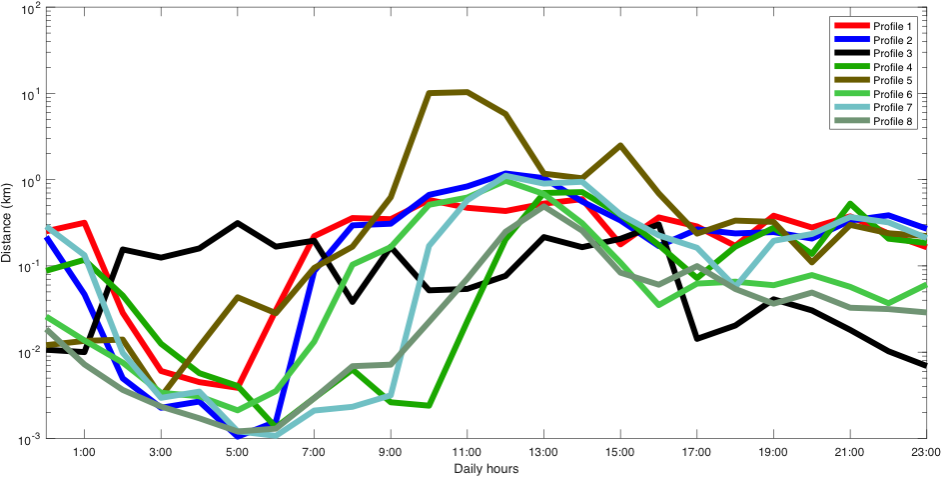
Distance traveled profiles of patient A.

She participated in the study from April 6, 2017 to February 28, 2018 and owned a Samsung Galaxy S7 that ran Android 6.0.1. Figure 3 shows that the MDL criterion selected 8 different clusters (ie, types of days or mobility patterns). We plotted the patient’s inferred mobility patterns (in logarithmic scale), which are given by the mean of each cluster. For instance, profile 5 corresponded to a more active day and, on the days associated with this profile, the patient was more active between 9:00 and 16:00. Moreover, some of these profiles reported similar activity variations throughout the day. The sleep period was identified by a decrease in the activity between 1:00 and 6:00.

**Figure 4:**
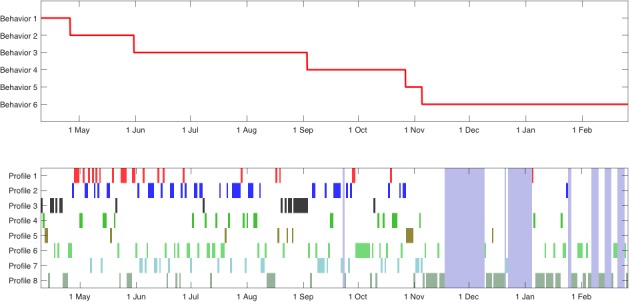
Representation of mobility pattern changes (upper) identified by the technique and corresponding patterns (lower) during study participation of patient A.

Figure 4 shows the output of the second step of the proposed method, the change-point detector; this figure displays the dates of the change-points (top) and the classification of each day given by the clustering technique and its temporal evolution (bottom). The algorithm identified a few dates as mobility pattern changes. Concretely, changes were noted on April 26, May 31, August 19, September 3, October 27, and November 5. These changes appeared when the probability (a portion of time) of each type of day varied.

Finally, we must point out that in Figure 4, where the temporal evolution of the types of days is shown, vertical light-blue rectangles indicate that the data corresponding to the marked days were completely missing. Even in these cases, the technique was robust enough to work properly.

Patient B was a 45-year-old woman. She was diagnosed with dysthymia and prescribed a daily oral medication of sertraline 100 mg. The clinical assessment of depression showed clinical improvement of depressive symptoms (June 7, 2017: PHQ-9=20; July 5, 2017: PHQ-9=8). Overall, medical records showed improvement during follow-up, explained by the participant as an improvement in cognitive performance, a decrease of death thoughts, and improvement of hedonic capacity.

**Figure 5:**
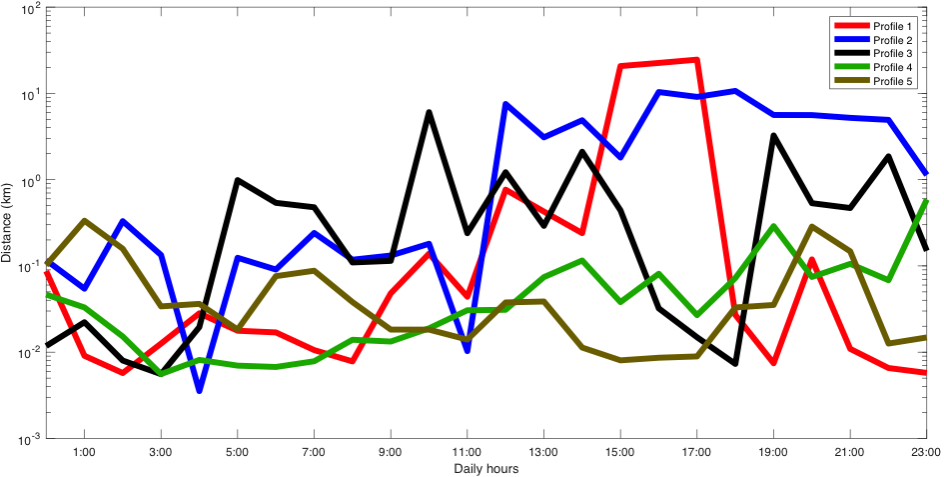
Distance traveled profiles of patient B.

**Figure 6:**
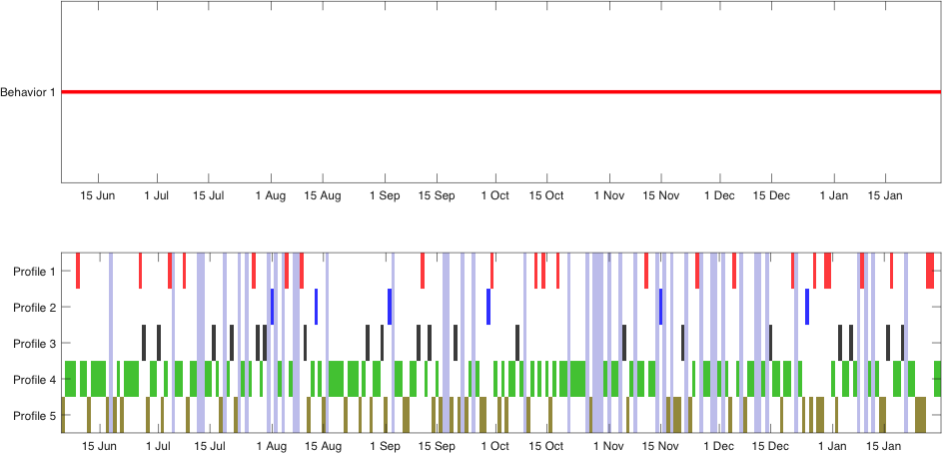
Representation of mobility pattern changes (above) identified by the technique and corresponding patterns (down) during study participation of patient B.

She participated in the study from June 7, 2017 to January 30, 2018, and owned a Samsung Galaxy A5 running Android 6.0.1. In this case, the technique selected 5 different clusters. Figure 5 shows the patient’s average mobility patterns. Figure 6 shows that our technique did not identify any change and that profile 4 was the most common, which was a low-mobility profile (there was not a single hour with >1 km). In this particular patient, clinical changes did not correlate with mobility as the main symptoms were expressed in cognitive and hedonic areas.

Patient C was a 40-year-old woman. She was diagnosed with a moderate depressive episode. She was prescribed a daily oral medication of paroxetine 20 mg, which was changed to vortioxetine 10 mg in August owing to the lack of improvement. Medical records showed an improvement after the change to vortioxetine.

**Figure 7:**
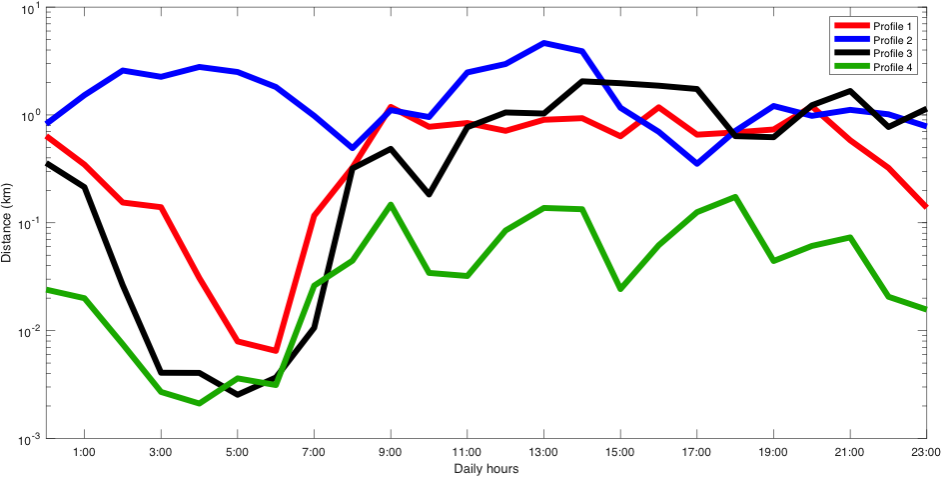
Distance traveled profiles of patient C.

**Figure 8:**
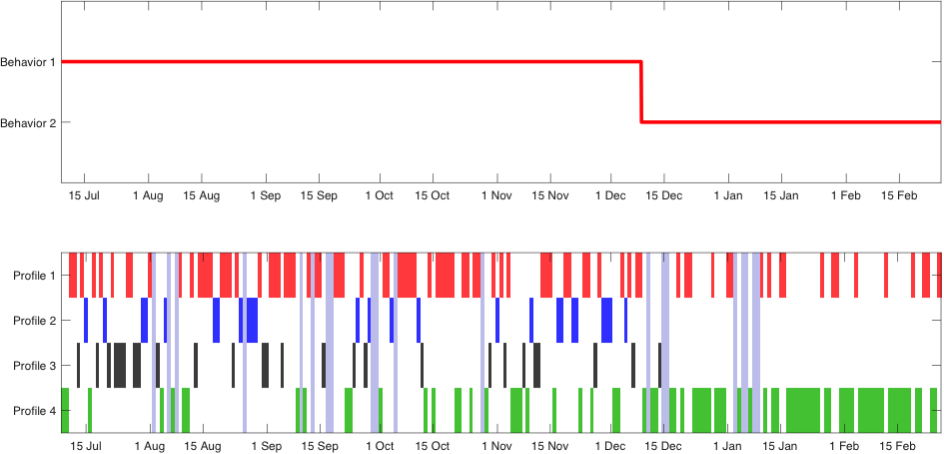
Representation of mobility pattern changes (above) identified by the technique and corresponding patterns (down) during study participation of patient C.

This patient participated in the study from June 9, 2017 to February 28, 2018, and owned a Samsung Galaxy A5 that ran Android 7.0. In this case, the technique only considered 4 different types of days. Figure 7 shows the average distance traveled in each cluster, where we observed that the patient was more active after 7:00 in 3 out of the 4 profiles. Moreover, the remaining profile, profile 2, showed increased activity during the night, and profile 4 corresponded to a low-mobility profile. Figure 8 shows that the change-point detection algorithm detected only one change on December 9; after this date, the low-mobility profile began appearing more often, which possibly indicated a decrease of the patient’s physical activity.

The clinical assessment of depression showed a decrease in depressive symptoms during the follow-up period (June 9, 2017: PHQ-9=22; Sept 9, 2017: PHQ-9=5; December 1, 2017: PHQ-9=4). Clinical improvement was associated with improved sleep time and sleep quality. A change of her work location led to less commuting, which can also explain the observed mobility patterns.

Patient D was a 36-year-old man. He was diagnosed with recurrent depressive disorder and prescribed a daily oral medication of venlafaxine retard 150 mg and lamotrigine 100 mg. He was included in the study after psychiatric hospitalization discharge, and clinical and functional remissions were observed in successive appointments in the outpatient setting. The clinical assessment of depression showed minor clinical improvement (March 17, 2017: PHQ-9=6; April 20, 2017: PHQ-9=2; May 24, 2017: PHQ-9=2; and June 26, 2017: PHQ-9=0).

**Figure 9:**
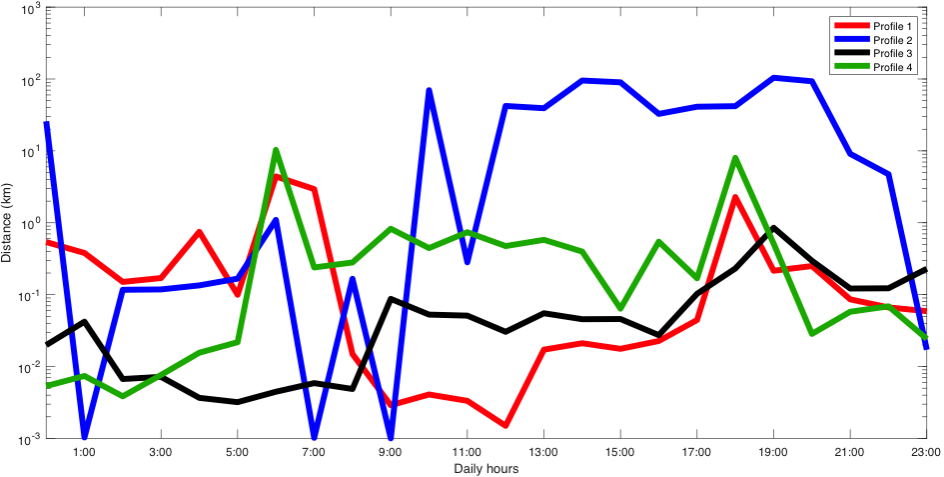
Distance traveled profiles of patient D.

**Figure 10:**
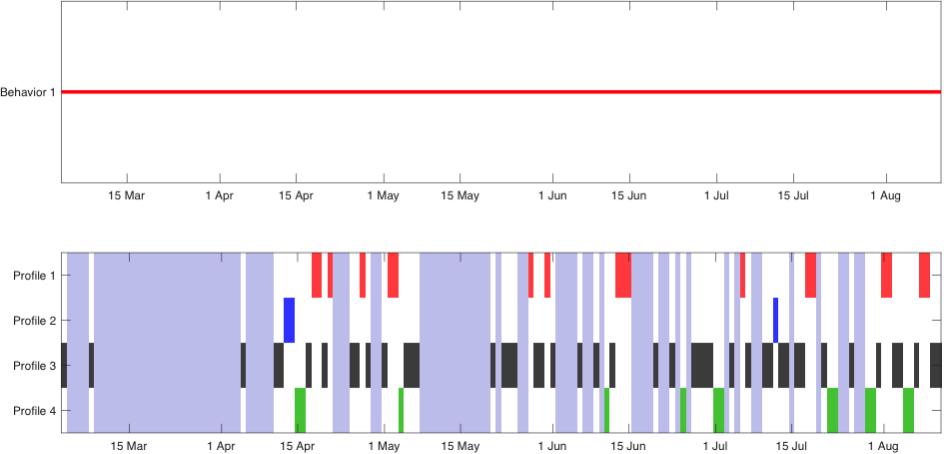
Representation of mobility pattern changes (above) identified by the technique and corresponding patterns (down) during study participation of patient D.

He participated in the study from April 6, 2017 to August 11, 2017, and owned a Samsung Galaxy J7 running Android 6.0.1. Figure 9 shows that the number of profiles selected by the MDL criterion was 4. Profiles 1, 3, and 4 corresponded to typical urban mobility profiles. Some showed higher mobility during day or night, and some had peaks at commuting times (7:00 and 19:00). However, profile 2 corresponded very likely to a trip as the average movement per hour was around 100 km. Figure 10 shows the results of the change-point detector, which did not detect any change-point; this is coherent with the clinical evolution of the patient.

Patient E was a 42-year-old woman diagnosed with adjustment disorder with depressed mood and lumbar stenosis. She was prescribed a daily oral medication of escitalopram 15 mg, pregabalin 150 mg, and ketazolam 15 mg, besides antialgic medication. Fluctuations in the mood level were observed during follow-up in relation to back pain exacerbation.

**Figure 11:**
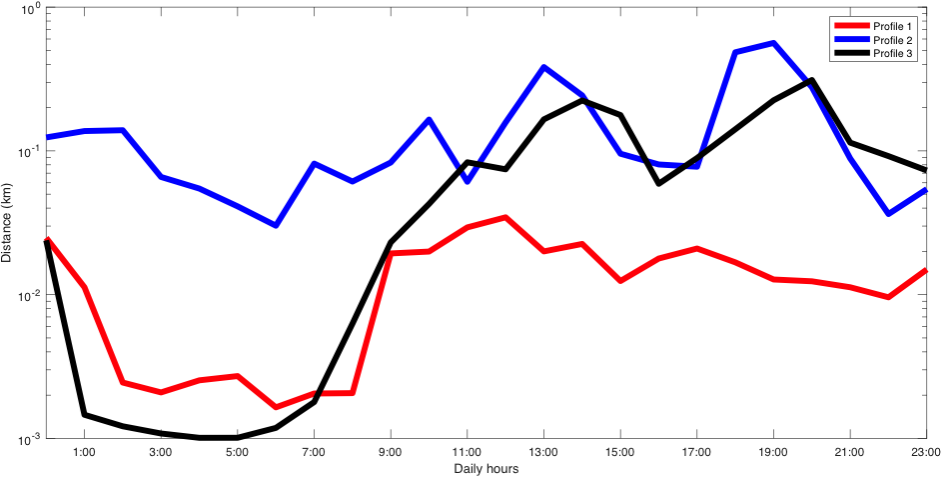
Distance traveled profiles of patient E.

**Figure 12:**
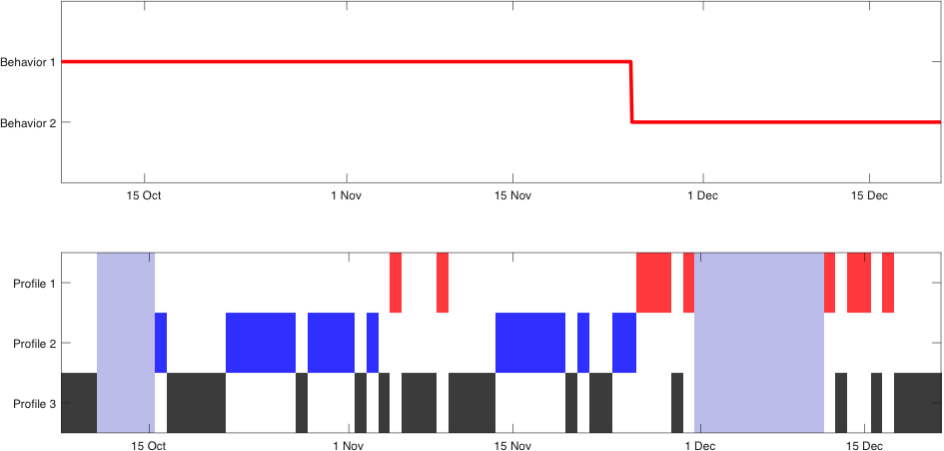
Representation of mobility pattern changes (above) identified by the technique and corresponding patterns (down) during study participation of patient E.

This patient participated in the study from October 11, 2017 to December 21, 2017, and owned a BQ Aquaris M5 that ran Android 6.0.1. In addition, this patient showed improvement in depression scores during the study (June 23, 2017: PHQ-9=10; October 5, 2017: PHQ-9=6). In this case, as Figure 11 shows, the MDL criterion only selected 3 profiles, as the amount of data was rather small and, otherwise, would very likely have resulted in overfitting. Overall, 2 profiles corresponded to activity during the daytime, whereas profile 2 showed activity evenly distributed during the whole day. Figure 12 shows that the technique identified one change-point on November 25, 2017. Interestingly, this change-point appeared when profile 2 disappeared. The change-point coincided with an increase of painful osteoarticular symptoms.

## Discussion

### Principal Findings

This study showed that the eB^2^ system was capable of identifying mobility pattern changes, which may be used as proxies for behavioral changes and relapses. The technique was composed of 2 parts—a clustering algorithm to learn mobility profiles, which was based on a mixture of Gaussians model, and a change-point detector to identify probability changes of the mobility patterns. It is important to point out that detecting changes from one type of day to another does not suffice, what matters are the probability changes because we could have a type of day given by a typical workday and another one given by a typical weekend day; however, the change from the former to the latter (or *vice versa*) should not have been identified as a mobility pattern change.

This pilot study showed that the proposed technique could aid clinicians to detect relapses and other clinical changes. However, before its use in a clinical setting, the changes identified by the algorithm need to be interpreted. In this paper, we have shown the results from a few selected cases that may illustrate the potential applications of the eB^2^ system in the outpatient follow-up of patients with depressive disorders.

The (possible) behavioral changes identification technique proposed in this study was based on the unsupervised processing of data from smartphone sensors. In particular, this work focused on detecting mobility pattern changes, which could be used as indicators of behavioral changes and only exploited the GPS location data. The reasons were two-fold: (1) it yielded a relatively simple algorithm and (2) it admitted an easy clinical interpretation (more or less related to physical activity). However, as we have previously pointed out, the platform captured much more data, and the technique can be adapted also to exploit these additional data.

Our final goal would be the identification of more general behavioral changes (eg, Web-based social interaction) in outpatients, which has important applications for a wide range of chronic conditions, including mental health disorders. Apart from the continuous assessment of bioparameters themselves, smartphone-based monitoring would also allow researchers to gather information on context and environment, which may prove valuable for the interpretation of the monitored biomedical data (eg, information about weather conditions) and allow for a better interpretation of changes.

### Clinical Contextualization of Smartphone Data

When the changes identified by eB^2^ were contextualized in a given patient’s routine, we were able to extract valuable information related to clinical changes. Thus, in our 5 selected patients, we identified different profiles of activity.

Interestingly, changes and different profiles represented different clinical scenarios. For instance, patients B and D showed no changes, whereas for patient A, the changes corresponded to a worsening. The algorithm detected this worsening on April 26, 2017 when the PHQ-9 depression score increased between April 6, 2017 and May 31, 2017. This participant did not show up for follow-up in September, although she continued using the eB^2^ app and we cannot, therefore, establish clinical correlations from there on. Incidentally, a change-point was detected on September 1, 2017, which may be related to the drop-out from the follow-up. In patient D, the absence of changes reflected minimal clinical changes and stability in symptoms. However, patient B was an example in which mobility patterns were not useful for clinical purposes, as the proposed method did not identify any change, but there was, indeed, a clinical improvement. In this particular patient, the remaining data collected by the smartphone might be more useful, but this analysis is out of the scope of this work.

In addition, changes could represent both improvement and worsening, depending on the specific patient. On the one hand, the change identified on December 9, 2017 for patient C corresponded to a clinical improvement owing to the disappearance of increased activity during the night from that date onwards, reflecting a better night’s sleep. In addition, a profile with low activity started to appear more often and, in fact, at this moment, the patient started to have a quiet lifestyle. In contrast, for patient E, a change represented a clinical worsening owing to the emergence of a profile of less activity and the disappearance of a profile of daytime activity. Both the emergence and disappearance of the above profiles indicated the worsening of the patient’s condition owing to the exacerbation of her back pain.

Overall, these results highlighted that apps, such as eB^2^, can be used for personalized psychiatry and that we are witnessing a paradigm shift from the traditional identification of shared factors in mental illnesses to individual and unique characteristics for each patient, that is, personalized medicine. A study presented EMA as the future of outpatient follow-up [18]. However, this technique strongly relied on patients’ participation and was, therefore, prone to missing data [19].

### Limitations

This study was conducted on a limited sample of patients with a limited time scale. Thus, it did not allow for the complete identification of ADLs; only mobility patterns could be identified. In addition, we did not have access to an ecological self-reported description of the patients’ behavior. Ecological data are usually based on self-assessments and provide information that may be correlated with the digital phenotyping [19]. Ideally, we should have combined self-reported ecological data capture [20] with the results obtained by the eB^2^ system to test whether the automatically detected changes correlated with the clinically diagnosed changes or data ecologically reported by patients. In this study, the algorithm detected changes in mobility patterns, which could be identified as behavioral changes. However, in this explanatory setting, we were not able to completely determine whether these behavioral changes identified by the algorithm corresponded to a clinical modification or the emergence of any normal or abnormal behavior. Moreover, we identified several factors that may explain the changes and which were not related to any modification in depressive symptoms. Furthermore, this study was based only on GPS data and many patients disabled this sensor; this is a problem that we will need to address in the future, and it is, therefore, important to convince patients not to disable the location in their smartphones. Nonetheless, location is not the only source of information, albeit it is important, and we should consider other types of data in future studies.

Data privacy is a serious concern in the eHealth research area. The eB^2^ app captured data from smartphones, which possibly was a deterrent for patients to accept the app [21]. However, the selected patients were aware of the general approach of our method and were not very concerned about sharing their personal data as it was anonymized in the smartphone. Another major concern regarding personal electronic data is data security [22]. To uphold patients’ privacy and reduce the risk associated with nonlegitimate access, all the sensible information stored in the eB^2^ server was hashed and anonymized. Concretely, phone numbers, email addresses, Bluetooth, and Wi-Fi Media Access Control addresses were hashed using the SHA-1 algorithm, and the location was transformed using a noninvertible function. Specifically, we stored randomly rotated relative location coordinates, where the origin was the location that was most common during the first 3 days after installation (typically patients’ home). Our app was (and still is) available through app stores, such as Google Play or App Store, which allowed us to continuously update and improve the app based on newly discovered bugs and also user feedback. For instance, we have improved the battery consumption, which should improve patient adherence in the future.

### Future Application

Smartphone-based systems for managing and monitoring behavior present a highly promising field of innovation in health care. The normal use of a smartphone on a daily basis generates a larger amount of data than the amount that is typically collected in questionnaire-based studies or Web-based interventions; however, it requires that patients carry their smartphone most of the time. A smartphone sensor-based analysis already showed interesting results in the assessment of bipolar disorder [13], depression symptoms [8], prediction of schizophrenia [15], and sleep duration [23]. This work is in line with recent proposals of Torous et al., who established digital phenotyping as a promising method in the assessment of patients with mental health conditions [24].

We proposed a preliminary assessment of a method for patients with mental health conditions. Our system was able to identify changes in the mobility patterns of outpatients, which may correlate with behavioral changes and relapses. In the future, eB^2^ may also be used for the assessment of physical activity in therapeutic programs or the identification of ADLs in the elderly [6].

### Conclusions

We have developed a system that can capture data from the smartphone’s native sensors and other wearables. The eB^2^ system is composed of a smartphone app and a backend server. The preliminary results of the ongoing eB^2^ study showed the feasibility of an unsupervised detection method for detecting mobility pattern changes, which we considered proxies for behavioral changes, in outpatients by exploiting the data acquired by the eB^2^ app. So far, only location data were used, which resulted in relatively simple processing techniques and allowed for an easy clinical interpretation of the results. Of note, this method did not need intervention from patients. However, it was crucial that patients carried their phone all the time. With the development of the eB^2^ system, we aimed to address most challenges raised by eHealth technologies in ecological monitoring.

## Abbreviations

ADL: activities of daily living
API: application programming interface
eB ^2^: Evidence-Based Behavior
eHealth: electronic health
EMA: ecological momentary assessment
GPS: global positioning system
MDL: minimum description length
PHQ-9: Patient Health Questionnaire-9
